# Genome Sequence of *Leuconostoc mesenteroides* subsp. *cremoris* Strain T26, Isolated from Mesophilic Undefined Cheese Starter

**DOI:** 10.1128/genomeA.00485-14

**Published:** 2014-06-05

**Authors:** T. B. Pedersen, W. P. Kot, L. H. Hansen, S. J. Sørensen, J. R. Broadbent, F. K. Vogensen, Y. Ardö

**Affiliations:** a Department of Food Science, Faculty of Science, University of Copenhagen, Copenhagen, Denmark; b Department of Biology, Faculty of Science, University of Copenhagen, Copenhagen, Denmark; c Department of Nutrition, Dietetics, and Food Sciences, Utah State University, Logan, Utah, USA; d Department of Environmental Science, Aarhus University, Roskilde, Denmark

## Abstract

*Leuconostoc* is the main group of heterofermentative bacteria found in mesophilic dairy starters. They grow in close symbiosis with the *Lactococcus* population and are able to degrade citrate. Here we present a draft genome sequence of *Leuconostoc mesenteroides* subsp. *cremoris* strain T26.

## GENOME ANNOUNCEMENT

Here we present a draft genome sequence of *Leuconostoc mesenteroides* subsp. *cremoris* strain T26, which was isolated from a traditional mesophilic undefined cheese starter culture (1). This species commonly accounts for 2 to 10% of the starter population, which is predominantly *Lactococcus lactis*. Through its heterofermentative metabolism and ability to degrade citrate, *L. mesenteroides* subsp. *cremoris* contributes to the eye and aroma formation in Gouda type cheeses (2). Currently there are two *L. mesenteroides* subsp. *cremoris* genome sequences publicly available, those of strain 19254^T^ (GenBank accession number ACKV00000000) and strain TIFN8 (3). Both of these strains were also isolated from dairy starter cultures. The draft genome of strain T26 has a size of 1,833,933 bp, with an average G+C content of 38.4%. A sequencing library was prepared using the Nextera XT (Illumina, USA) kit according to the manufacturer’s recommendation as 2 × 250-base paired-end reads using the Illumina MiSeq (Illumina, USA) technology, followed by sequencing, as a part of the flowcell, using the MiSeq (Illumina, USA) technology. Reads were trimmed and assembled with the CLC Genomics Workbench 6.5.1 (CLC bio, Denmark). Resulting contigs were annotated using the RAST server (4).

Two complete prophages were found, together with other phage remnants. The heterofermentative metabolism and ability to degrade citrate were confirmed with the finding of genes coding for key enzymes in these two pathways. We have analyzed *L. mesenteroides* subsp. *cremoris* T26 for specific cheese-related enzyme activities (1), and future work will involve comparative genomics with other publicly available *Leuconostoc* genomes.

### Nucleotide sequence accession numbers.

This whole-genome shotgun project has been deposited at DDBJ/EMBL/GenBank under the accession number JAUJ00000000. The version described in this paper is version JAUJ01000000.
